# COVID-19 infection across workplace settings in Qatar: a comparison of COVID-19 positivity rates of screened workers from March 1st until July 31st, 2020

**DOI:** 10.1186/s12995-021-00311-5

**Published:** 2021-06-17

**Authors:** Mohamed Ghaith Al-Kuwari, Asma Ali Al-Nuaimi, Jazeel Abdulmajeed, Sandy Semaan, Hamad Eid Al-Romaihi, Mujeeb Chettiyam Kandy, Selvakumar Swamy

**Affiliations:** 1 Primary Health Care Corporation, Doha, Qatar; 2 Ministry of Public Health, Doha, Qatar

**Keywords:** COVID-19, Occupational health, Occupational risk, Infectious disease, Workers

## Abstract

**Introduction:**

COVID-19 transmission was significant amongst Qatar’s working population during the March–July 2020 outbreak. The study aimed to estimate the risk of exposure for COVID-19 across various workplace settings and demographics in the State of Qatar.

**Methods:**

A cross-sectional study was conducted utilizing surveillance data of all workplaces with 10 or more laboratory-confirmed cases of COVID-19. These workplaces were categorized using a mapping table adapted from the North American Industry Classification System (NAICS) codes, 2017 version. The data was then analyzed to estimate and compare the positivity rate as an indicator of the risk of developing COVID-19 infection across various workplace settings in the State of Qatar.

**Results:**

The highest positivity rate was reported amongst the Construction & Related (40.0%) and the Retail & Wholesale Trade sectors (40.0%), whereas, the lowest positivity rate was attributed to the healthcare workplace setting (11.0%). The highest incidence of COVID-19 infections occurred in South Asian nationalities and in the male gender. The private funded sector employees have seen higher positivity rate than employees of the governmental funded sector.

**Conclusion:**

The elevated risk of infection in Construction and Retail & Wholesale Trade is probably due to environmental and educational vulnerabilities. The predominant labor force of those workplace categories is South Asian craft and male manual workers. Alternatively, the better containment of the healthcare workplace setting can be attributed to the enforcement of infection control and occupational safety measures. These findings imply the importance of using preventive and surveillance strategies for high-risk workplace settings appropriately.

## Introduction

The current COVID-19 outbreak that emerged in Wuhan City, Hubei Province, China [1], represents one of the most challenging public health threats globally faced. On January 30th, 2020, the World Health Organization (WHO) declared COVID-19 a public health emergency of international concern [2]. The second week of July 2020 had seen more than 13 million confirmed cases across 215 countries [3].

The initial stage of the epidemic in the State of Qatar started on February 29th, 2020, with a COVID-19 positive citizen case traveling back from Iran who had directly been isolated upon his arrival. Additional infected citizens traveling back to Qatar were immediately isolated to avoid Community spread. On March 11th, the state of Qatar witnessed a sudden surge of 226 locally transmitted new cases in 1 day, which entails an outbreak with localized transmission, where sporadic infections with the pathogen occur. On May 22nd, the Ministry of Public Health declared that the State of Qatar had entered the peak phase of the pandemic represented by a widespread human infection. By the second week of July, Qatar had recorded more than 109,000 confirmed COVID-19 patients for a total of 2.7 million inhabitants [4].

A major route of COVID-19 transmission has already been identified as the workplace setting [5]. The association between workplace site exposure and the disease is significant: the first documented case was working in a seafood wholesale market in Wuhan [6]. Additionally, it has been officially declared as an occupational disease in countries like South Africa and Canada when it is considered the result of occupational exposure. Germany and Italy have also declared COVID-19 an occupational disease but only limited to the healthcare sector [7].

Moreover, several research papers have been published illustrating the prevalence of exposed workers in the healthcare industry [8, 9]. According to preliminary data from China, healthcare workers (HCWs) facing COVID-19 represent a high-risk category [10, 11]. The Occupational Information Network (O*NET) in the United States has developed a COVID-19 Occupational Risk Score to determine which occupations face the highest risk of exposure to COVID-19 based on three criteria: contact with others, physical proximity, and exposure level. The O*NET risk scores place healthcare workers, paramedics, and flight attendants in the high-risk categories [12]. Although healthcare workers are exposed to a particular risk of infection because of the nature of their work, other workplace settings would also have an increased risk for COVID-19 contamination because of the environment they work in and the continuity of their work during the pandemic; This includes other essential workers, front line workplace settings, food-related workplace settings and any work that requires interactions at proximity [13].

Qatar’s working population represents 76.95% of the country’s total population. It is predominantly constituted of Non-Qatari laborers who represented 95% of the total labor force in 2018. Additionally, a further breakdown of the working population displayed a male majority of 86% and a relatively young age group of 20–44 years of age representing 81% [14].

During the first wave of the COVID-19 pandemic, the Government has strategized and implemented restrictions to curb the COVID-19 spread. Widespread testing and regular screening had been executed and recommended by the Ministry of Public Health (MoPH) for public-facing workplace sectors such as the Accommodation and Food workplace category [15]. Surge plans had been developed and implemented across the healthcare sector to manage increased demand for testing as per the epidemic dynamic [16].

The Government has allowed partial functioning of all workplaces, yet, many workplace settings remained relatively fully operational, which highlights the importance of understanding the burden of COVID-19 at the workplace as well as its parameters.

In this study, we aim to estimate the risk level of exposure to COVID-19 at various workplace settings in the state of Qatar through the comparison of respective positivity rates. The study will also estimate how occupational risks of exposure to COVID-19 vary across sociodemographic characteristics. Indeed, the pandemic has revealed that the occupational factor of risk of exposure to COVID-19 is strongly associated with educational and socioeconomic status, which can contribute to higher rates of infection [17].

The findings may be used to better target interventions aimed at decreasing risks of transmission of infectious diseases such as COVID-19 during different phases of an epidemic, including the progressive lifting of emergency measures mandating workplace closures.

## Methodology

A cross-sectional study was conducted to analyze COVID-19 infections in workplaces using surveillance database available from the MOPH. The surveillance database aggregates patient laboratory data and the corresponding patient’s employment data to identify workplace clusters. One thousand eight hundred workplaces, identified as workplace clusters with 10 or more laboratory-confirmed cases of COVID-19 during the period March 1st - July 31st, were included for analysis. Any confirmed case not working in the identified company, aged below 18 years old, student, or retired, were excluded from the study. The researchers categorized these workplaces using a mapping table adapted from the North American Industry Classification System (NAICS) codes 2017 version [18]. The NAICS classifies businesses and industries into different levels of aggregation based on the economic sectors. In this study, the researchers used the broader NAIC categories (20 codes) and adapted it to Qatar’s economic and social contexts (11 codes) divided into two categories- public funded and private funded (Table 1).

**Table 1:** Workplace Classifications in the State of Qatar

Economic sector	Workplace Classification	Inclusions
Public Funded	National security	Armed forces, military and police
	Oil & gas	Companies specialized in oil and gas operations- energy upstream, midstream, and downstream.
	Public service	Various ministries and other entities offering public services
	Health care	Health centers, hospitals, private clinics, medical laboratories, and healthcare administrative offices
Private funded	Accommodation and food services	Restaurants, hotels and commercial residential complexes.
	Construction & related	Construction and contracting companies as well as manufacturers specialized in construction equipment and material.
	Finance & business	banks and financial institutions as well as private businesses offering consulting services or administrative support.
	Holding/conglomerate with diversified services	Holding companies offering more than one type of service. For the majority, Holdings offer real estate, construction, and retails services
	Retail and wholesale trade	Supermarkets, grocery stores, pharmacies as well as factories, manufacturers, and agriculture domain.^a^
	Support waste management and remediation services	cleaning and hospitality companies, private security services, and waste & facility management services
	Transportation and warehousing	Entities specialized in transportation logistics, warehousing and storage.

^a^Agriculture domain refers here to retails farms and farmers markets

Subsequently, the surveillance data was mapped to the list of categorized workplace settings. The final database created for this study consisted of patient demographics, workplace categories and COVID-19 laboratory results.

The compiled data extract was imported into STATA v 15.1 – (StataCorp. 2017. College Station, TX: StataCorp LLC.). The data was analyzed to estimate and compare the test positivity rate as an indicator for the risk of developing COVID-19 infection across various workplace settings. Chi–square test was used as appropriate; a *p*-value of < 0.05 was considered significant.

Individuals might have been tested several times, but only the first positive result for each individual was counted in spite of the number of tests the individual may have had.

The nationality of tested individuals was established based on the official identification state card, the card is issued for all the residents and nationals in Qatar. Nationalities were grouped into seven main ethnicity groups for analysis purposes (Middle Eastern, North African, Sub-Saharan African, South Asian, South East Asian, European, and others for American, Australian, New Zealander, and, Central & Western Asian. Demographic characteristics were derived from their electronic medical records and the age of the tested persons was categorized into five main groups (18–30, 31–40, 41–50, 51–60, > 60).

The compiled data extract was imported into STATA v 15.1 – (StataCorp. 2017. College Station, TX: StataCorp LLC.). The data was analyzed to estimate and compare the test positivity rate as an indicator for the risk of developing COVID-19 infection across various workplace settings. Chi–square test was used as appropriate; a *p*-value of < 0.05 was considered significant.

Workplace Classification monthly data for positivity rate has been compiled for each of the 11 sectors to form parabolic trends which highlight the impact of the COVID-19 throughout the period ranging from March to July 2020. Comparable trends were then grouped into 4 separate Graphs to call attention to similarities in the evolution of the epidemic for grouped sectors.

## Results

During the period ranging from March 1st to July 31st, 2020, a total of 201,006 individuals were tested, out of which 59,175 were found positive. This leads to an overall positivity rate of 29.4%.

With regards to age distribution, four of the five age groups that were taken into consideration, from 18 years to 50 years of age, have seen positivity rates ranging between 28.9 and 30.5%. The older age groups have recorded slightly lower positivity rates, at 27.3% for individuals between 51 and 60 years of age and 23.2% for individuals older than 60 (Table 2).

**Table 2:** Shows the positivity rate distribution across different age groups, genders, ethnicities, economic sectors, and workplace classifications, and highlights the statistical significance of the results using *P*-Value analysis. COVID-19 Positivity Rate Distribution (March 1st - July 31st, 2020)

–	Tested	Positive	Positivity Rate	*P*-Value
	201,006	59,175	29.40%	
Age group (years)				
18–30	60,006	18,201	30.30%	< 0.001
31–40	80,441	23,261	28.90%	
41–50	40,602	12,403	30.50%	
51–60	16,742	4563	27.30%	
> 60	3215	747	23.20%	
Gender				
Male	177,489	56,829	32.00%	< 0.001
Female	23,517	2346	10.00%	
Ethnicity				
Southern Asia^a^	130,720	47,728	36.50%	< 0.001
South East Asia^b^	23,528	3924	16.70%	
Northern Africa	19,311	3207	16.60%	
Middle East	11,907	1268	10.60%	
Sub-Saharan Africa	10,457	2670	25.50%	
Europe	2697	138	5.10%	
Other regions	2386	240	10.10%	
Economy Sector				
Private	126,848	46,541	36.70%	< 0.001
Public	74,158	12,634	17.00%	
Workplace Classification				
Accommodation and Food Services	6291	2083	33.10%	< 0.001
Construction & Related	51,832	20,708	40.00%	
Finance & Business	5352	1867	34.90%	
Health Care	23,253	2557	11.00%	
Holding/Conglomerate with diversified services	5686	1755	30.90%	
National Security	25,564	5545	21.70%	
Oil & Gas	9087	1677	18.50%	
Public Service	16,254	2855	17.60%	
Retail and Wholesale Trade	20,659	8261	40.00%	
Support Waste Management and Remediation Services	21,944	7573	34.50%	
Transportation and Warehousing	15,084	4294	28.50%	

South Asia include Afghanistan Bangladesh, Bhutan, Sri Lanka, India, Maldives, Nepal, Pakistan

South East Asia includes Brunei, Myanmar, Cambodia, Indonesia, Laos, Malaysia, Philippines, East Timor, Singapore, Vietnam, Thailand

When comparing between male and female genders, the disparity is clear, with 32.0% of all tested males recording positive results for COVD-19 infection, whereas 10.0% of females tested positive. This is in line with the overall positivity rate of 29.4%, seeing how the male population constitutes 88.3% of the total tested individuals and 96.0% of all tested positive (Table 2).

An ethnicity breakdown highlights that most of the population tested is Southern Asian (65.0%), recording a positivity rate of 36.5% and accounting for 80.7% of all accounted COVID-19 positive results. The African population recorded a positivity rate of 25.5%, excluding Northern Africa, which was more in line with Southeast Asia, as they saw respective positivity rates of 16.6 and 16.7%. The Middle East, Europe, and other regions- comprising of Oceania, Central & Western Asia, and the Americas, have had lower positivity rates of 10.6, 5.1%, and a cumulative 10.6%, respectively (Table 2).

The private sector, accounting for 78.7% of all tested positive, recorded a positivity rate of 36.7%. Alternatively, the public sector, comprised of the Public Service, National Security, Healthcare, and Oil & Gas categories, had seen a much lower cumulative positivity rate of 17% (Table 2).

Delving deeper into the different Workplace Classifications, we quickly note that the Construction & Related and Retail & Wholesale Trade Categories have the highest positivity rate of 40.0%. They are followed by the Finance & Business, Support Waste Management and Remediation Services, Accommodation & Food Services, Holding/Conglomerate with diversified Services, and Transportation & Warehousing categories with respective positivity rates ranging between 34.9 and 28.5%. Finally, as mentioned previously, the public sector categories recorded lower positivity rates with 21.7% for National Security, 18.5% for Oil & Gas, 17.6% for Public Service, and 11.0% for the Healthcare category, from which a total of 23,253 individuals were tested.

The *P*-Values corresponding to the analysis of the above five categories have all resulted in values less than 0.001, which highlights the significance of the different groups belonging to each category (Table 2).

### Workplace categories overall analysis

**Fig. 1:**
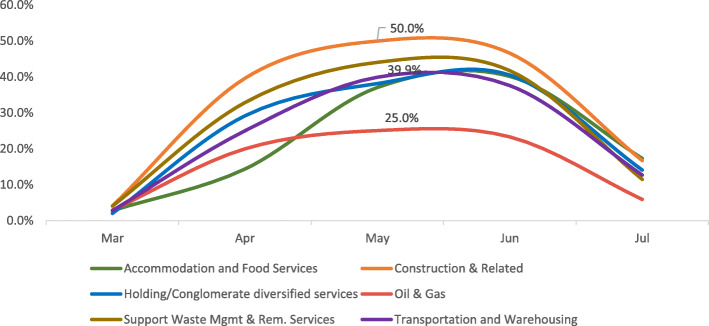
GROUP A monthly trend of infection

**Fig. 2:**
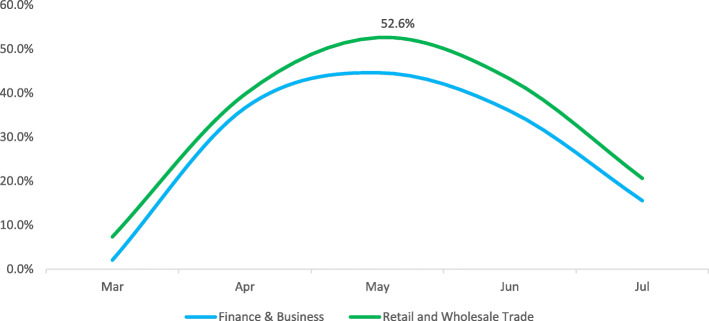
GROUP B monthly trend of infection

**Fig. 3:**
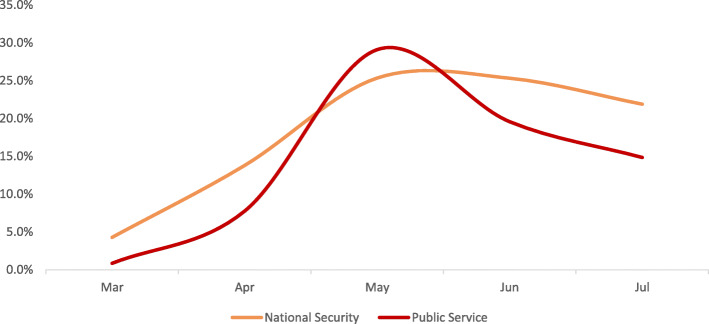
GROUP C monthly trend of infection

**Fig. 4:**
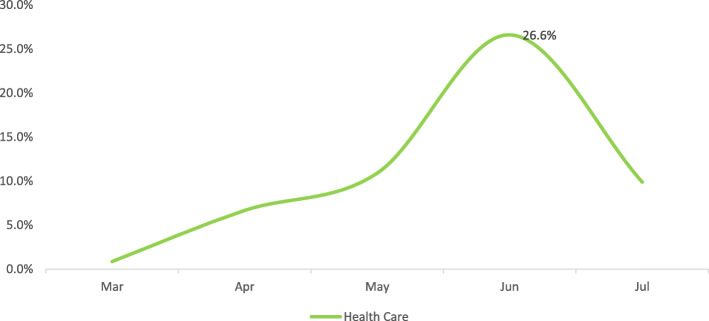
GROUP D monthly trend of infection

The Retail & Wholesale, and Construction & Related categories have recorded high positivity rates of 52.6 and 50.0% respectively at peak. Finance & Business, Support Waste Management & Remediation Services, Holding/Conglomerate with Diversified Services, Accommodation & Food Services, and Transportation & Warehousing categories have seen positivity rates ranging from 39.9 to 44.6% at peak, whereas, the Oil & Gas, National Security, Healthcare, and Public Service categories have had positivity rates ranging from 25.0 to 29.1% at peak (Figures. 1, 2, 3 and 4).

The workplace categories have then been grouped as per their epidemic trends.

Pattern A includes the following 6 categories - Construction & Related, Support Waste Management & Remediation Services, Transportation & Warehousing, Accommodation & Food Services, Holding/Conglomerate with Diversified Services, and Oil & Gas. those 6 sectors have followed the same trend with a rapid increase in positivity rates through March and April peaking in May/June and rapidly decreasing after June (Fig. 1).

Pattern B includes Retail & Wholesale Trade as well as the Finance & Business. Those categories have seen positivity rates rapidly increase from March to peak in May and then gradually decrease throughout June and July (Fig. 2).

Pattern C includes The National Security and Public Service. Categories whose positivity rates have gradually increased from March until reaching a peak in May and remaining relatively stagnant until July (Fig. 3).

Finally, the singular Pattern D which includes the Healthcare category. The Healthcare Category saw a slow increase from March extending unto May followed by a spike in June and a steep decrease after that (Fig. 4).

## Discussion

Qatar has taken general precautionary measures to prevent the spread of COVID-19. Parts of those measures were related to the workplace. They varied from having a complete shutdown (e.g., Public transportation, Education, and some type of retail stores) to a mandatory 80% workforce to work from home, applied to all other businesses [19]. The exception was the healthcare sector, national security, food industry, supermarkets, airport, and some major state construction projects that continued their work as usual. Additionally, the following categories were allowed to work remotely: employees over the age of 55 years, pregnant employees, and employees with chronic diseases related to cardiac, renal, cancer, diabetes, and hypertension.

The study revealed the highest positivity rate for both the retail and wholesale sector and the construction sector. The retail workplace setting, namely supermarkets, has been considered in the current outbreak as one of the occupational groups at risk of contracting COVID-19 disease at the workplace, given its public interaction and frontline focused nature of work [20]. Similarly, the Finance and Business sector, which includes banks and other business/ financial institutions, followed the same trend as grouped above due to the similar customer-centric approach and the same implemented restrictions as the retail sector.

Although construction sites have no direct public exposure, such as in retail and wholesale, the high number of cases might be related to environmental and educational factors. It has already been demonstrated how workers can be exposed to several hazards at a time as multiple elements in different areas can interact with each other, resulting in a cumulative exposure that can affect and increase the worker’s overall risk [21]. At the start of the outbreak, there was no available educational material translated in the languages of the workers coming mostly from Southern Asian countries. Also, Craft and Manual Workers (CMWs), predominantly of the male gender and belonging to a relatively young age group, live in crowded dormitory-type shared accommodation, are usually transported in buses at full capacity, and, are therefore in constant proximity of one another [22]. They also often gather for social and recreational activities, shared dining, and use of shared equipment e.g. kitchen appliances, with minimal compliance to social distancing requirements. This environment ultimately increases the likelihood of COVID-19 transmission amongst them [23]. The accommodation type was considered in Qatar as one of the strong forecasters and substantial contributing risk factors for health problems amongst migrant workers [24].

Taking a closer look at the workplace classifications which are bundled under group A for relatively following the same trend of infection, it would be interesting to note that all six sectors rely heavily on CMWs which could explain the similar positivity rate patterns. Alternatively, the public sector, namely the Public Service, National Security, and Healthcare sectors, with the exception of the Oil and Gas sector which partially follows a construction aspect, rely less on CMWs due to the administrative nature of the works which require more advanced educational qualifications. In fact, all four public funded sectors have seen lower positivity rates, both in sharpness of trends and in magnitude. This ultimately ties back to the disparity in educational and socio-economic backgrounds. A cohort study conducted in the United States, aimed to quantify the associations between socio-economic status and COVID-19–related cases, found that lower education levels are strongly associated with higher rates of COVID-19 cases [25].

Although the health care sector has been considered as a workplace with a high risk for occupational exposure to the infection, Healthcare had the lowest overall positivity rate amongst all other categories. It might be attributed to the enforcement of infection control and occupational safety measures such as continuously wearing masks, frequent handwashing, and constant availability of sanitizers. Health authority has also put in place a range of teleconsultation services that have proven quite significant in emergency responses, to deliver care while reducing the risk of contamination [26]. Periodic testing and isolation could also be one of the possible explanations for the low positivity rate associated with the slowly increasing trend of the health care sector for the months of March up to May. As part of the Qatar National COVID-19 Action Response Plan, a contact tracing strategy was implemented promptly at the beginning of the rise in cases. Contact Tracing and Case Investigation teams traced close contacts who were then tested and isolated as per the country quarantine guidelines and WHO close contact criteria. The government had put in place a national target aiming that 90% of contacts are traced and assessed within 24 h from the confirmation of the positive case. This identification and follow-up of contacts were implemented for all sectors, prioritizing high-risk settings such as healthcare facilities [27]. It has been demonstrated that rapid tracing and testing of close contacts is effective to identify and isolate secondary positive cases more rapidly and can prevent onward transmission of the pandemic [28].

The country increased its healthcare capacity and was able to perform up to 20,000 polymerase chain reaction (PCR) tests per day which is substantial given the size of Qatar’s population [29].

The sharp increase in positivity rates in May, peaking in June, can be explained as a reaction to almost all other sectors peaking in May which increased the risk of infection and overall exposure for HCWs during that period.

A study highlighting Work-related COVID-19 transmission in six Asian countries, stressed the importance of work-related transmission of COVID-19 outbreak outside healthcare settings such as transportation or retail settings. Also, the proportion of healthcare workers (HCWs) among locally transmitted cases was smaller than non-HCWs in the included countries/areas [30]. Those findings most probably underline the efficacy of the use of Personal Protective Equipment such as n95 masks, gloves, eye protection, screening, and knowledge about the pandemic in healthcare settings. HCWs are also strongly supported by international institutions to prevent and contain any outbreak within healthcare facilities: The World Health Organization has developed several specific guidance documents regarding COVID-19 for HCWs, including rights, roles, and responsibilities with key considerations for safety and health. They have also established a risk assessment tool that is to be used by health care facilities to determine the risk of infection of all HCWs who have been exposed to a COVID-19 patient [31].

A study conducted at Cambridge University Hospitals NHS Foundation Trust has presented findings from a systematic staff screening program for more than 1000 asymptomatic HCWs in their workplace, in addition to more than 200 symptomatic staff or household contacts. The data has demonstrated the utility of comprehensive screening of HCWs with minimal or no symptoms. This approach is estimated to be critical for protecting patients and hospital staff and the containment in the transmission of COVID-19, especially that it included many asymptomatic cases [32].

In addition, tracking back the infection source in healthcare settings is also more straightforward, and thus containment is smoother. It is, therefore, crucial to protect essential workers not working in healthcare settings because their risk of infection is often under-estimated especially compared to the healthcare sector, and, their employers might not always provide adequate Protective Equipment training or screening [33].

Qatar had begun lifting restrictions in a four-phased approach that started in June. The systematic testing of specific employees who were intended to return to work such as workers employed in restaurants and in the accommodation industry and the contact tracing strategy can explain the sharp increase in cases in the Public and Accommodation & Food industries during the same period. The screening initiative enabled the isolation of COVID-19 cases before the eventual opening of public services, hotels, and restaurants to the public. In fact, looking at positivity rates for all work categories in Groups A, B, and C, we can highlight that, during the months of June, all trends were pointing towards a steady decrease in numbers of infections.

The study has some limitations. Seeing how the state of Qatar’s healthcare system is predominantly public, it is important to note that the healthcare sector, in this study, includes a minor proportion of private facilities which have been accounted for under the public umbrella to simplify the workplace sector segregation. Although the government has implemented regular mandatory screening for specific workplaces such as the Public sector as mentioned above, the information regarding the frequency of testing at other workplaces such as the Construction category is unavailable. Despite these limitations, the used database and cumulative records included all PCR testing done in workplaces in Qatar, as representative of the real population.

In Conclusion, these findings highlight the importance of appropriately implementing strategies of prevention and surveillance as well as the adherence to a communications approach at the workplace to ensure conveying consistent health messages that are easy and accessible for all segments of the population for an optimum health outcome. Furthermore, socio-economic factors should be considered when implementing public health interventions to ameliorate the disparities in the impact of COVID-19 on distressed communities.

## Data Availability

The raw data supporting the conclusions of this article will be made available by the authors, without undue reservation.
